# Measuring Mentalizing Ability: A Within-Subject Comparison between an Explicit and Implicit Version of a Ball Detection Task

**DOI:** 10.1371/journal.pone.0164373

**Published:** 2016-10-10

**Authors:** Annabel D. Nijhof, Marcel Brass, Lara Bardi, Jan R. Wiersema

**Affiliations:** Ghent University, Ghent, Belgium; Ecole Normale Superieure, FRANCE

## Abstract

The concept of mentalizing has been widely studied, but almost exclusively through tasks with explicit instructions. Recent studies suggest that people also mentalize on a more implicit level. However, to our knowledge, no study to date has directly contrasted the effects of implicit and explicit mentalizing processes on an implicit dependent measure within-subjects. We implemented this by using two versions of an object detection task, differing only on secondary catch questions. We hypothesized that if explicit mentalizing relies on complementary processes beyond those underlying implicit mentalizing, this would be reflected in enhanced belief effects in the explicit version. Twenty-eight healthy adults watched movies in which, during the first phase, both they themselves and another agent formed a belief about the location of a ball, and although irrelevant, these beliefs could influence their ball detection reaction times in the second phase. After this response phase, there were occasional catch questions that were different for the explicit and implicit task version. Finally, self-report measures of autism spectrum disorder (ASD) symptomatology were included, as the literature suggests that ASD is related to a specific deficit in implicit mentalizing. Both in the explicit and implicit version, belief conditions had a significant effect on reaction times, with responses being slower when neither the participant nor the other agent expected the ball to be present compared to all other conditions. Importantly, after the implicit version, participants reported no explicit mentalizing awareness. In our neurotypical sample, ASD symptoms were not found to correlate with either explicit or implicit mentalizing. In conclusion, the reaction time patterns in the explicit and implicit version of the task show strikingly similar effects of mentalizing, indicating that participants processed beliefs to the same extent regardless of whether they mentalized explicitly or implicitly, with no additional effects for explicit processing.

## Introduction

Theory of Mind (ToM; [1]), also called mentalizing, refers to the ability to attribute mental states (such as beliefs, desires and intentions) to oneself and others. It is needed in order to understand and predict other people’s behavior, and therefore it is thought to underlie all human social interaction [2]. The initial tests that were developed to measure ToM ability are false-belief tests such as the Sally-Anne task [3]. In these tasks participants are required to explicitly reason about the beliefs of another agent. Studies have shown that explicit mentalizing is cognitively demanding, as increased cognitive load and impaired executive functioning interfere with explicit belief reasoning [4–6]. Children are able to perform well on these mentalizing tasks only from about four years of age onwards [7]. Because of this, ToM was thought to be an ability that requires explicit reasoning, that is cognitively demanding and appears relatively late in a child’s development. However, two recent lines of research, discussed below, have questioned these features of ToM, which in turn has led to the claim that humans can also mentalize implicitly. An implicit form of mentalizing is thought to develop early and to be fast, cognitively efficient but inflexible, whereas explicit mentalizing processes would develop later and would be slower, more deliberate and flexible, but therefore also more cognitively demanding [8]. One line of argument for the existence of an implicit form of mentalizing comes from developmental studies. Several researchers have shown, by means of eye-tracking, that children show signs of tracking other people’s beliefs at a much younger age than four years [9–13]. In the study by Kovács and colleagues, infants as young as seven months old showed evidence of processing the true or false beliefs of an agent in a movie, since these beliefs affected their looking times to an object [13]. Given that these infants were obviously not instructed to track others’ beliefs, this can be taken as initial evidence for the existence of implicit mentalizing processes alongside, or instead of, the more established explicit mentalizing. In their study, the same paradigm was also applied to a group of adults, who showed a similar effect; in their case it was indicated by reaction times to the (expected or unexpected) presence of the object. In a different study, adults showed implicit processing through their eye movement patterns as well [14]. A second line of argument against a solely explicit definition of mentalizing can be found in the literature on autism spectrum disorder (ASD). The socio-communicative problems associated with this developmental disorder have often been explained in terms of deficits in ToM [15–17]. Still, two findings in the ASD population are inconsistent with this ToM deficit explanation, but the existence of implicit mentalizing may explain these inconsistencies. Firstly, explicit ToM ability develops around the age of four years [3], yet autistic symptoms are present in children at a much younger age [18]. Therefore, the socio-communicative impairment in ASD cannot exclusively be explained by an explicit ToM deficit. Implicit mentalizing is thought to be present at birth or to appear very early in development [8,19], hence a deficit in implicit mentalizing may explain early observed impairments in ASD [20]. Secondly, traditional ToM tests, such as first- and second-order false-belief tasks, are often passed by high-functioning children and adults with ASD (see [21], for an overview), despite the fact that they do experience severe difficulties with social cognition and mentalizing in daily life. Their successful performance on false-belief tests has been claimed to be dependent on compensatory strategies [22,23]. As implicit mentalizing is thought to act fast and inflexibly, it would offer fewer possibilities for such strategies. Indeed, recent findings in adolescents and adults with ASD suggest that ASD is related to a deficit in implicit mentalizing, even in the absence of explicit mentalizing problems [24–27]. This brings up an important point about the nature of implicit and explicit mentalizing processes. If a deficit in implicit mentalizing is possible without a deficit in explicit mentalizing, this seems to be in line with the suggestion that humans have two distinct systems for mentalizing ("two-system account"; [8,19,28]), and that both would have their own, perhaps partially overlapping, underlying neural architecture [29]. Still, studies investigating implicit mentalizing processes, and their similarities and differences to explicit processes, are relatively scarce, and from the above findings the question arises of whether there really are two separate systems for mentalizing. An alternative hypothesis, and one that we would like to investigate further in the current study, is that the mentalizing system is in principle implicit, and that on explicit tasks people simply recruit additional, non-mentalizing processes such as executive functioning and/or language skills [30], depending on the specific task requirements. This is also in line with the hypothesis that people with high-functioning ASD use their executive functioning skills to solve explicit ToM tasks in test situations, thus circumventing their mentalizing deficits [31]. In order to investigate whether two separable forms of mentalizing exist, what processes make them unique and during what kinds of tasks they are involved, research directly contrasting explicit and implicit mentalizing is needed [21]. The aim of the current study was to do just this, by comparing the effects of using implicit and explicit mentalizing processes directly, on the same (implicit) dependent variable and using a within-subjects design, thus circumventing the limitations of previous studies of implicit mentalizing. An important limitation is that most studies to date tested only implicit mentalizing and made no comparison with explicit mentalizing (e.g. [13,32]). Other studies did compare implicit and explicit mentalizing, but with tasks that were different in terms of the nature of the tasks themselves and/or the stimuli that were used (e.g. [24,25,27]), making it difficult if not impossible to attribute a differential pattern of results to the mere addition of explicit processes. In recent years, some attempts have been made to contrast implicit and explicit mentalizing on more comparable tasks [33,34], or even on the same dependent variable [35], and all these studies interpreted their results as evidence for the existence of two separate kinds of mentalizing.

However, for different reasons the findings from these studies are not conclusive. The different task versions were not compared within-subjects [34,35], or outcome measures of both versions were different and no debriefing was used to look into participants’ solution strategies [33], so that it cannot be taken for granted that they did not process the implicit task explicitly. In the study by Van der Wel and colleagues [35], just as in the current study (although between-subjects), one and the same implicit outcome variable was used to measure both implicit and explicit belief processing. Participants needed to follow the location of an object in a movie. Another agent present in the movie also held a (similar or different) belief about the object location, but only in the explicit group participants were asked to keep track of this. At a cue, participants had to move their mouse cursor to the location where they thought the object was, and in addition to reaction times, their cursor movement trajectories were used as a dependent variable. The authors found that both implicit and explicit belief processing influenced participants’ continuous movement trajectories, but incongruence of the participant’s and agent’s beliefs slowed down reaction times in the explicit group only. However, only for this group, and not for the implicit group, they added a second measure: participants sometimes had to indicate where the other agent thought the relevant object was. This dual-task situation may have resulted in greater cognitive demands for the explicit group, and thus, with the absence of a secondary task in the implicit group, an imbalance between the two that cannot be explained solely in terms of explicit mentalizing processes. In the current study, we used a paradigm that we developed (the ‘Buzz Lightyear task’), that is an adaptation of the paradigm developed by Kovács and colleagues [13] and has already been validated for use both in patient and non-patient groups [36]. We created two fully comparable versions of a task that implicitly measures mentalizing: the general task (ball detection) was always the same, just as the dependent variable (reaction time to the ball), which, importantly, was always measuring belief processing only implicitly. By adding different catch questions, belief processing was either made explicit or remained implicit, so that we will still speak of having created an ‘implicit and explicit version’. Additionally, a debriefing session was included to ensure that participants were unaware of the task purpose (measuring the degree to which they processed another agent’s, Buzz’, beliefs) in the implicit version of the task. We tested the two versions within-subjects, in a sample of neurotypical adults. In addition to comparing the implicit and explicit processing of beliefs, we tested the relationship between the measures of implicit and explicit mentalizing, and symptoms of ASD. As discussed above, it has been claimed that ASD is related to a specific deficit in implicit mentalizing [26]. In fact, a recent study [36] that applied the same implicit mentalizing task as the one used in the current study, revealed that participants with ASD with more severe autistic symptomatology and social difficulties, were also more impaired in implicit belief processing. The question that we wanted to address here is whether these findings hold for ASD symptoms in the neurotypical population. Therefore, we took a dimensional approach and related measures of ASD symptomatology in our neurotypical sample to both implicit and explicit mentalizing findings, to further our insights into the association between ASD symptoms and mentalizing. We predicted that participants would track the belief of the agent implicitly, as would be reflected by the implicit dependent measure (their ball-detection reaction times). In line with the findings of Kovács and colleagues [13], we expected that responses to the ball would be slower in the condition in which neither the agent nor the participant had expected it than in the condition in which only the agent had expected the ball, indicating mentalizing took place (referred to as the ToM index). It could be that the effects of processing an agent’s beliefs are the same, whether they are processed implicitly or explicitly, because belief calculation can be done entirely through implicit processes. In this case, similar condition effects are expected for both versions. Alternatively, however, if by asking about beliefs explicitly additional (explicit) processes are recruited that may be helpful in the mentalizing process, the critical effects on the implicit measure can be expected to be stronger for the version with explicit catch questions. In light of the recent debate about the two-system account of mentalizing that Apperly and Butterfill [8] proposed, it would be informative to see if the extent to which participants showed evidence of mentalizing when they were asked to do so explicitly, was related to when they were not. Therefore, correlations were calculated between individuals’ measures of mentalizing on the implicit and explicit versions of the task. Finally, elevated ASD symptomatology was expected to be associated with lower measures of implicit belief processing, while the link between ASD symptoms and explicit mentalizing was expected to be smaller or even absent.

## Materials and Methods

### Participants

Thirty-seven participants (11 male, mean age: 23.7 years, SD: 3.4 years, three left-handed), with no reported history of psychiatric or neurological disorders, participated in the study. They were recruited through the university and received payment after their participation. All of the participants gave written informed consent prior to the study. The study was approved by the local ethics committee of the Faculty of Psychology and Educational Sciences of Ghent University. Six participants did not perform significantly above chance level on either one or both types of catch questions (three on implicit, one on explicit, two on both), and were therefore excluded from further analysis. Additionally, three participants were excluded because during the debriefing they revealed explicit awareness on the implicit task. Data analysis was thus carried out on data of 28 participants (9 male, mean age: 24.2 years, SD: 3.4 years, three left-handed). Questionnaire scores were obtained for 25 of 28 participants in a separate session.

### Tasks

The tasks were presented using Presentation software, version 16.5.

#### General task

**Fig 1:**
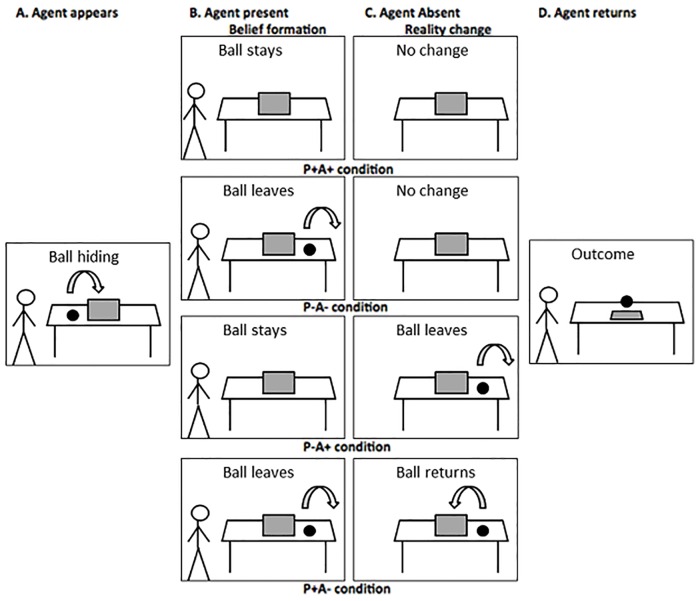
Schematic illustration of the eight conditions, resulting from four different options in the Belief Formation phase (B and C), and two options in the Outcome phase (D). In the first part of the movie (A), the ball rolls behind the screen. In the second part (B), Buzz is present and the ball sometimes changes location. In the third part (C), Buzz is absent and the ball sometimes changes location. In the last part (D), the occluder falls and the ball is present or not. Note: these are not the original stimuli, as these could not be printed due to copyright issues.

Participants watched short (13,850 ms) video animations of 720 by 480 pixels. In each video, an agent (*Buzz Lightyear*) placed a ball on a table. The ball rolled behind an occluder and subsequently there were four possible continuations (see Fig 1, Belief Formation phase):

1. Resulting in the agent holding a true belief (i.e., true in the eyes of the participant) about the ball being present (P+A+ condition: P = participant, A = agent, + = belief of presence, − = belief of absence).
2. Resulting in the agent holding a true belief about the ball being absent (P-A- condition).
3. Resulting in the agent holding a false belief about the ball being present (P-A+ condition).
4. Resulting in the agent holding a false belief about the ball being absent (P+A- condition).

In each video, the agent left the scene at some point. This varied in timing between conditions: Buzz left 5000 ms after movie onset for condition P-A+, 7624 ms after movie onset for condition P+A-, and 9874 ms after movie onset for conditions P+A+ and P-A-. In order to ensure that participants were paying attention to the on-going video, they had to press a key (‘X’ on the keyboard) when Buzz left the scene. The agent always returned to the scene at 12,694 ms.

In the Outcome Phase (see Fig 1), the occluder fell (at 13,250 ms). Participants had to press a key (‘B’ on the keyboard) as quickly as possible when the ball was behind the occluder, which was the case on half of the trials. The absence or presence of the ball was completely random and independent of the belief formation phase. It could thus be expected or unexpected for the participant and/or the agent. Sometimes a catch question was presented after the end of the movie (see next section), and before the next movie there was always an ITI (black screen) of 1000 ms.

Thus the design consisted of three factors with two levels each: 2 (participant’s belief: present/absent) x 2 (agent’s belief: present/absent) x 2 (outcome: present/absent). Reaction time (RT) data were only available for conditions with outcome ‘present’. Movies for each condition were repeated 10 times. These 80 movies per task version were presented in a randomized order in two blocks of 40 trials, with a short break in between blocks. Before the start of the actual experiment, both on the implicit and explicit versions of the task, participants completed four practice trials. During these trials they received feedback, while during the real experiment they did not. No catch questions were presented after practice trials.

#### Catch questions

The implicit and explicit versions of the task only differed with respect to the catch questions. These questions appeared randomly after 20% of the movies: 8 per 40 trials of each block in both task versions. Questions were presented in black text on a light grey background for 1000 ms. In the implicit task, the question was: ‘Did Buzz have a blue cap?’ The cap could be either blue (50% of the movies) or red (50%). In the explicit task, the question was: ‘Did Buzz think the ball was behind the screen?’ Participants were explicitly instructed to keep track of Buzz’ initial belief about the location of the ball, that is, prior to the revelation of its true location. The answer to this catch question was also ‘Yes’ in 50% of the movies. It can be assumed that if participants performed above chance on these catch trials, they were consciously keeping track of the agent’s belief during the movies, and that mentalizing on this version of the task was therefore explicit. The words ‘Yes’ and ‘No’ were presented on the left or right of the screen in both task versions. 50% of catch questions had ‘Yes’ printed left and ‘No’ right, 50% vice versa. In this way, responses could not be planned in advance. Participants had to respond to the answer on the left with their left middle finger (‘W’ on the keyboard), to the answer on the right with their left index finger (‘X’ on the keyboard).

### Questionnaires

In order to measure ASD symptomatology, two questionnaires were administered: the Autism Spectrum Quotient (AQ) and the Social Responsiveness Scale—Adult version (SRS-A). The AQ [37] is a self-report screening questionnaire for adults, quantifying the degree to which one has those traits that are associated with the autism spectrum. It consists of 50 items and is divided into five subscales with 10 items each: social skill, attention switching, attention to detail, communication and imagination. Participants answer to each item on a scale of 1 to 4. The SRS-A [38] is a questionnaire measuring different behavioral dimensions that are characteristic of ASD. It primarily addresses social responsiveness in adulthood, by means of 64 items. These can be divided over four scales: social awareness, social communication, social motivation and rigidity/repetitiveness. Participants answer to each item on a scale of 1 to 4.

### Procedure

The experiment consisted of two sessions. During the first session, the implicit and explicit versions of the task were administered. This took about 30 minutes in total. During the second session, participants filled in the two questionnaires. This took about 15 minutes in total. Participants always started with the implicit version of the task. After completion, participants filled in a debriefing form based on the one used by Schneider and colleagues [27], which was adapted to the current task and translated to Dutch. It consisted of five questions (see S1 Appendix—Debriefing Form for an English translation). Then, participants continued with the explicit version. In order to encourage motivation, participants were told before the experiment took place that they could get a monetary bonus for their participation if they would belong to the fastest 25% of participants. They always received this bonus.

### Statistical analyses

A 4-by-2 repeated measures ANOVA with factors condition (P+A+, P+A-, P-A+, P-A-) and version (implicit, explicit) was carried out in order to test for effects on the reaction times, followed by a planned comparison between the crucial conditions P-A- and P-A+. A similar ANOVA was carried out for the number of misses as well as for the number of false alarms.

## Results

All statistical analyses were conducted with IBM SPSS Statistics 20 (SPSS Inc., Chicago, IL, USA).

### General task

Before starting the analysis of the ball response data, we performed an outlier analysis, removing responses per participant that were more than 3 standard deviations above or below the participant’s overall mean, or that were less than 100 ms. This resulted in a loss of 41 data points over all participants, but control analyses revealed that the results without having removed outliers were highly similar to the results described below.

#### Reaction time

Mean RT to the ball in the implicit version was 332 ms, and 329 ms in the explicit version.

**Fig 2:**
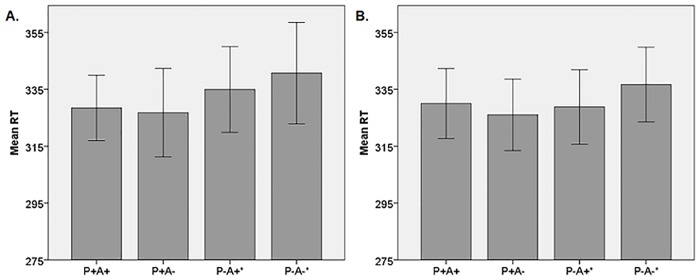
Mean reaction times per condition per task version, error bars represent +/- 2 standard errors. **A**: Implicit version. **B**: Explicit version. *: the difference between conditions P-A- and P-A+ is the ToM index.

We performed a 4 x 2 repeated measures ANOVA on reaction times, with within-subjects factors of condition (P+A+, P+A-, P-A+, P-A-) and version (implicit, explicit). Fig 2 displays the condition means per version. A significant effect of condition on RT was found (F (3, 81) = 6.55, p = 0.001). There was neither a significant effect of version (F (1, 27) = 0.43, p = 0.52), nor a significant effect of condition x version (F (3, 81) = 0.80, p = 0.50). The planned comparison between conditions P-A- and P-A+ revealed that participants were significantly slower on P-A- than on P-A+ (the ‘ToM-index’) (p = 0.02; see Table 1). As this comparison was carried out based on an a-priori hypothesis informed by the study of Kovács and colleagues [13], in order to preserve statistical power no Bonferroni correction was applied. For the sake of completeness, the results of the other comparisons are also shown in Table 1, but note that in order to correct for multiple comparisons, we should test these against a p-value of (0.05/6) = 0.0083 (Bonferroni correction). Only the comparison between condition P-A- and conditions P+A- and P+A+ were found to be significant after this correction (p = 0.002 and p = 0.004 respectively), in line with findings of Kovács and colleagues [13].

**Table 1:** Overview of the statistical results.

Condition 1	Condition 2	Mean Diff. (1–2)	Std. Error	p-value
*P-A-*	*P-A+*	*6*.*81*	*2*.*73*	*0*.02*
	P+A-	12.27	3.64	0.002*
	P+A+	9.44	2.98	0.004*
P-A+	P+A-	5.46	2.32	0.03
	P+A+	2.63	2.70	0.34
P+A-	P+A+	-2.83	2.87	0.33

*Note*. P = participant, A = other agent, + = belief of ball presence, − = belief of ball absence.

The planned comparison P-A- vs. P-A+ is displayed in italics.

* indicates a significant effect.

Running the analyses separately per task version gave similar results: the effect of condition was significant in the implicit version (F (3, 81) = 4.13, p = 0.01) as well as in the explicit version (F (3, 81) = 3.50, p = 0.02). Finally, we correlated the size of the crucial difference or ‘ToM index’ (i.e., the average difference in RT between P-A- and P-A+) in the implicit version with that in the explicit version. This correlation appeared not to be significant (r = -0.02, p = 0.93).

#### Accuracy

On average, across the two versions, participants missed the ball (or were too slow) on 3.6% of the trials, and hit the ‘B’ when the ball was not there (i.e., made ‘false alarms’) on 3.1% of the trials. A 4 x 2 repeated measures ANOVA on the percentage of misses, with condition (P+A+, P+A-, P-A+, P-A-) and version (implicit, explicit) as factors, showed a trend toward a significant effect of condition (F(3, 81) = 2.67, p = 0.07; Greenhouse-Geisser corrected). Mean percentage of misses was higher in the P-A- condition (M = 5.18%) than in the other three conditions (P+A+: 3.57%, P+A-: 2.86%, P-A+: 2.68%), indicating belief processing. The interaction effect between condition and version was not found to be significant (F(3, 81) = 1.47, p = 0.24; Greenhouse-Geisser corrected). When the same ANOVA was performed on the percentage of false alarms, it also revealed a trend toward a significant effect of condition (F (3, 81) = 2.40, p = 0.07). Mean percentage of false alarms was lower in the P-A- condition (M = 1.79%) than in the other three conditions (P+A+: M = 3.75%, P+A-: M = 4.11%, P-A+: M = 2.68%), again indicating belief processing. The interaction effect between condition and version was not found to be significant (F(3, 81) = 2.06, p = 0.13; Greenhouse-Geisser corrected).

### Catch questions

Mean RT on implicit catch questions was 880 ms, on explicit catch questions 731 ms. Participants were significantly slower on implicit catch trials (t(27) = 5.71, p < 0.001). On average, participants answered 84.1% of the implicit catch questions correctly, and 87.9% of the explicit catch questions. This difference in percentage of correct answers was not significant (t(27) = 1.587, p = 0.124). We can thus assume that participants were indeed explicitly tracking the belief of the agent in the explicit version, and that in terms of accuracy they performed as well on the catch trials in the implicit version as on those in the explicit version.

### Questionnaires

AQ and SRS-A scores, obtained from 25 of 28 participants, ranged between 2 and 25 for the AQ (mean score: 14.4, SD: 7.1), and between 12 and 95 for the SRS-A (mean score: 39.5, SD: 22.0). AQ and SRS-A scores were strongly positively correlated (r = 0.77, p < 0.001), as could be expected [39]. We correlated scores on both questionnaires with the size of the ToM index (the difference between P-A- and P-A+) in the implicit and explicit task version separately. The correlation between AQ scores and the ToM index was not significant, neither for the implicit (r = 0.20, p = 0.32) nor for the explicit version (r = 0.16, p = 0.44). SRS-A scores were also not significantly correlated with the ToM index (r = -0.07, p = 0.76 for the implicit version; r = 0.16, p = 0.45 for the explicit version).

## Discussion

In recent years, evidence for an implicit form of ToM has been accumulating, and this has been taken as indirect evidence for a two-system account of mentalizing as proposed by Apperly and Butterfill [8]. However, direct comparisons of implicit and explicit mentalizing are scarce. Therefore, if mentalizing problems can be solved at an implicit level, it is still unclear what difference it would make to use explicit processing instead of, or on top of the implicit mentalizing processes. The current study was intended to make such a direct comparison, by testing the effects of implicit and explicit processing of beliefs on the same implicit dependent measure, using a within-subjects design.

Importantly, our results support findings from previous studies in showing that participants implicitly calculated and sustained the beliefs of another agent. That is, overall, reaction times to the ball were slower in the condition where both participant and agent did not expect the ball, than in all other three conditions. Furthermore, the reaction time pattern was the same regardless of whether participants explicitly processed the agent’s beliefs (because of the explicit catch questions in the ‘explicit version’) or not (in the implicit version). This could be expected when assuming that belief calculation can be done entirely through implicit processes, and that if there were any additional processes recruited in order to answer the explicit catch questions, these did not affect the mentalizing process per se. Crucially, the debriefing procedure revealed that in the implicit version of the task, participants indeed were not consciously tracking the beliefs of the other agent. As for the explicit version, although we cannot say with certainty that participants did track Buzz’ beliefs explicitly and on-line, the combination of their relatively fast RTs and high level of accuracy in responding to the explicit catch questions strongly suggests that they did already create this explicit representation on-line. Thus, although we used a completely different stimulus set, we were able to replicate the pattern of results found in the study by Kovács and colleagues [13], and extend it to a version of their task in which participants had to answer explicit questions about Buzz’ belief. There was a slight difference to their results: the results seem to suggest that RTs for condition P-A+ were slower than for condition P+A-, a difference that Kovács and colleagues [13] did not find. Kovács and colleagues claimed that own belief and agent’s belief had a similar effect on RTs. We did not have an a-priori prediction about this comparison, hence the effect should be interpreted with caution (also given that it did not survive correction for multiple comparisons), but it could reflect a self-bias in how strongly beliefs are represented. Most importantly, we found the presence of the ToM effect both under implicit and explicit belief processing: the P-A- condition was significantly slower than all other three conditions. Results on the number of misses and false alarms participants made in each condition seem to reflect belief processing as well: on average, participants made most misses and fewest false alarms in the condition where both they and the other agent had not expected the ball. It was surprising to find that participants were faster in responding to explicit catch questions than to implicit catch questions. This could suggest a higher cognitive load in responding to the color of Buzz’ cap than to his belief, although this seems counterintuitive and also there were no differences in accuracy. Alternatively, it could be explained in terms of an order effect, as the implicit task version was always presented first and participants had to get used to the unexpected nature of the catch trials. The strikingly similar pattern of results for both the explicit and implicit version suggests that if any additional processes are at play during explicit mentalizing compared to implicit mentalizing, these do not have an influence on the implicit measure of mentalizing. However, the finding that there is a complete lack of a correlation between the implicit and explicit ToM index is rather surprising. This could mean that in the two versions, participants did rely on two different systems but still showed similar reaction time patterns. Rosenblau and colleagues [33] also reported an absence of correlation between scores on an implicit and explicit task, but in contrast to our study, they used different outcome measures for the two tasks. Of course, one cannot draw strong conclusions based on the absence of a correlation as this can be due to several reasons, it may for instance also be explained by a magnitude variability within subjects for explicit and/or implicit ToM processes. A neuroimaging approach is needed in order to know if the same or different brain regions are activated during the implicit and explicit versions of the current task. Kovács, Kühn, Gergely, Csibra and Brass [40] investigated implicit mentalizing at the neural level, by means of fMRI, with a version of the Buzz task slightly different from the one used in the current study, and found that the spontaneous tracking of the beliefs of another agent was related to activations in right temporo-parietal junction (rTPJ) and medial prefrontal cortex (mPFC), brain areas known to be consistently activated during explicit mentalizing [41,42]. This finding seems to indicate that implicit and explicit mentalizing processes are in fact more similar, and underlying brain regions more overlapping, than would be assumed based on a strict ‘two-system account’ of mentalizing (see also [30]). However, in their study no direct comparison was made with an explicit version of the task; such a direct comparison at the neural level is needed in order to find conclusive evidence. Finally, we did not find a link between the ToM index and ASD symptomatology, neither for the explicit nor for the implicit ToM index. This is not in line with the findings reported by Deschrijver and colleagues [36], who did report that individuals with more ASD symptoms showed a smaller ToM index of implicit mentalizing. They did however find such a link within a group of people with a formal diagnosis of ASD, while in the current study the association with ASD symptoms was tested in a neurotypical population. The fact that we did not find a correlation may be due to restricted variance (either in mentalizing ability, presence of ASD symptoms, or both) in our neurotypical sample. Alternatively, it could be that no relationship between measures of ASD symptoms and ToM measures exists in a non-clinical population, which would suggest a categorical difference between people with and without ASD. More research is warranted to address this issue. We are aware of the recent critique [43] on the implicit task developed by Kovács and colleagues [13]. The authors raised concerns about the processes underlying the condition effects in the original Kovács study, as they conclude that these effects are fully explained by inconsistencies in the timing of the attention check (that is, the response to the agent leaving the scene). For the movies used in our study, apart from the fact that they were accelerated (lasting 13,250 instead of 18,500 milliseconds), timing was exactly as in the study by Kovács, Téglás and Endress [13]. This would mean that similar concerns about the timing of the attention check may apply to our study and findings. However, in our opinion, it is unlikely that differences in timing of the attention check can (fully) explain the condition effects (significant ToM index) as found in the current study. Firstly, as a check within our own data, we correlated within-participants the RT to the attention check and the ball detection RT for the conditions on which the calculation of the ToM index (P-A+ and P-A-) was based. In line with the reasoning of Phillips and colleagues [43], it would be expected that when the response to the first event comes later in the movie, that the response to the second event will be later as well, due to the ‘quick succession’, especially in the P-A- condition, with the shortest interval between these events. However, no such correlation between RT to the attention check and ball detection RT was found, neither for the P-A+ nor for the P-A- condition (P-A+: average Z = 0.081, P-A-: average Z = 0.066; average correlation over all participants, computed using a Fisher’s r-to-Z transformation). As Phillips and colleagues [43] mention, in their results with the same paradigm there is not only a difference between P-A+ and P-A-, but also between P+A+ and P+A-. They explain this ‘cross-over effect’ (the slower responding in P+A+ and P-A- conditions) as due to the attention check being later in P+A+ and P-A- than in P+A- and P-A+ conditions, leading to both slower responding in the P+A+ condition relative to P+A- and slower responding in condition P-A- relative to P-A+ (the ToM index). Crucially, however, no cross-over interaction pattern is observed in the results of the current study. The expected ToM index is observed; but following the logic of Phillips and colleagues [43] one would also expect reaction times to P+A+ to be slower than to P+A-, which was not the case, neither in the implicit, nor in the explicit task version. Hence, the RT pattern in the current study does not reflect this cross-over effect. Secondly, Phillips and colleagues find a possible explanation for the influence of the timing differences on the results in terms of differences in refractory period. Indeed, research has shown that there is an influence of a short stimulus onset asynchrony (SOA) on the reaction time to a second stimulus, referred to as ‘psychological refractory period’ (PRP; see e.g., [44]). However, this PRP only has a short-term effect, usually lasting up to maximally several hundred milliseconds [44,45]. In the movies of the current study, the shortest SOA between S1 (‘agent leaves’) and S2 (‘ball appears’) encountered is 3.376 seconds (in the P+A+ and P-A- condition), which seems far beyond the reach of a PRP effect. Thirdly, following the reasoning of Phillips and colleagues [43], one would assume one single factor (namely, the timing of the attention check) to contribute to the ToM index of both the implicit and explicit task version. Thus, the fact that no correlation was found between the ToM index (P-A- minus P-A+) in the implicit and in the explicit task, is also unexpected from their point of view. Finally, as mentioned previously, Deschrijver and colleagues [36] carried out the same paradigm (implicit version only) in a group of adults with autism spectrum disorder (ASD). The size of individuals’ ToM index was found to correlate with ASD symptom severity in the ASD group. That is, the difference between P-A+ and P-A- was smaller for people with more self-reported symptoms of ASD, suggesting, as hypothesized, less implicit mentalizing in people with more symptoms of ASD. Again, this seems hard to reconcile with the claim of Phillips and colleagues [43] that the ToM index does not reflect mentalizing but is fully due to the timing of the attention check. Of course, none of these arguments can be taken as definite proof against the claims of Phillips and colleagues, and further research is warranted to more systematically investigate this issue. However, we believe that taking these arguments together it is unlikely that timing differences of the attention check can fully explain our results. Most importantly, the lack of correlation between interval timing and RTs in our own data forms a strong argument against a pure ‘timing explanation’ of our results, as it suggests that in the current study the interval differences did not confound the reaction time pattern. In conclusion, in this study we contrasted implicit and explicit mentalizing applying a within-subjects design, using highly comparable versions of a task differing only in terms of catch questions, and applied a debriefing procedure in order to make sure that participants had not been mentalizing explicitly during the implicit task version. Results seem to show that participants do use implicit mentalizing. Moreover, the pattern of results for implicit mentalizing was strikingly similar to that of explicit mentalizing. This suggests that participants could process beliefs to the same extent, whether they were doing this explicitly or implicitly, and that there are no additional effects for explicit belief processing, at least at the behavioral level. Although we do not believe that the current findings can be (fully) explained by difference in timing of the attention check, further research is warranted to systematically investigate this issue. In addition, future neuroimaging studies are needed in order to get more insight into the neural processes underlying both explicit and implicit mentalizing, and how similar or different they are.

## Supporting Information

S1 Appendix Debriefing Form (translated from Dutch). (DOCX)
