# Supplementary material for: Measuring Mentalizing Ability: A Within-Subject Comparison between an Explicit and Implicit Version of a Ball Detection Task
Source: PLoS One. 2016 Oct 10;11(10):e0164373. doi: 10.1371/journal.pone.0164373 (PMC5056736; doi:10.1371/journal.pone.0164373)
Supplement: S1 Appendix — (DOCX) [file pone.0164373.s001.docx]

**Appendix. Debriefing Form (translated from Dutch)**

1. What do you think the goal of this experiment was?
2. What do you think we wanted to study with this experiment?
3. Did you notice anything unusual about the movies?
4. Did you notice any particular pattern or theme to the movies?
5. What were you trying to do while watching the movies? Did you have any particular goal or strategy?
